# Mifepristone inhibits hepatoma growth by enhancing the GR-HSP60-survivin interaction to facilitate survivin degradation

**DOI:** 10.7150/jca.86611

**Published:** 2023-09-18

**Authors:** Ya-Hui Huang, Kwang-Huei Lin, Ming-Wei Lai, Chau-Ting Yeh

**Affiliations:** 1 Liver Research Center, Chang Gung Memorial Hospital, Linkou Main Branch, Taoyuan, Taiwan.; 2 Department of Biochemistry, College of Medicine, Chang-Gung University, Taoyuan, Taiwan.; 3 Research Center for Chinese Herbal Medicine, College of Human Ecology, Chang Gung University of Science and Technology, Taoyuan 333, Taiwan; 4 Division of Pediatric Gastroenterology, Department of Pediatrics, Chang Gung Memorial Hospital Linkou Main Branch, Taoyuan, Taiwan.; 5 Molecular Medicine Research Center, Chang-Gung University, Taoyuan, Taiwan.

**Keywords:** mifepristone, HSP60, survivin, glucocorticoid receptor, hepatocellular carcinoma

## Abstract

Silencing of heat shock protein 60 (HSP60) suppresses the growth of hepatocellular carcinoma (HCC). Mifepristone inhibits *HSP60* mRNA expression in *Chlamydophila*-infected epithelial cells. The aim of this study was to determine whether mifepristone could inhibit the growth of HCC cells by affecting the functions of HSP60. The effect of mifepristone on cell viability was examined by flow cytometry and a cell proliferation assay. Protein-protein interactions were examined using the immunoprecipitation assay. The anti-tumor effect of mifepristone was evaluated using a xenograft model. Our results indicated that mifepristone induces cell cycle arrest at the G1 phase and early-stage apoptosis in HCC cells. Instead of reducing the total amount of HSP60, mifepristone induced the release of mitochondrial HSP60 into the cytosol by causing a loss of ΔΨm, thereby enhancing glucocorticoid receptor (GR)-HSP60-survivin complex formation as well as survivin degradation. Animal models have confirmed the growth inhibitory effects of mifepristone on HCC, including changes in the abundance of HSP60 in mitochondria and cytosol, decreased survivin and Ki-67-positive cells, as well as increased cell apoptosis. In conclusion, the inhibition of HCC growth by mifepristone may be achieved by altering the subcellular distribution of HSP60 to enhance the formation of cytosolic GR-HSP60-survivin complexes in the cells, leading to the degradation of survivin.

## Introduction

Although there are many treatment options available for hepatocellular carcinoma (HCC), the disease continues to be one of the deadliest cancers worldwide. Typically, HCC patients are often diagnosed at an advanced stage, leading to a 5-year survival rate of approximately 12% 1. As a result, the development of novel and effective strategies or techniques for HCC treatment remains a crucial area of focus for oncologists and clinical researchers.

Our previous study demonstrated that silencing heat shock protein 60 (HSP60 or HSPD1) in HCC cells leads to a reduction in cell viability by inhibiting cell proliferation and inducing apoptosis. This effect is accompanied by a decrease in survivin expression. We found that HSP60 and survivin interact in the cytosol of HCC cells, and their expression levels are positively correlated in HCC tissues 2. Survivin, a molecule commonly overexpressed in HCC tissues, plays a role in promoting cell proliferation 3. To further confirm the growth inhibitory effects of HSP60, we delivered antisense HSP60 into mice, which resulted in reduced survivin expression in xenograft tumors 2. These findings suggest that HSP60 may represent a potential therapeutic target for HCC. Additionally, in 2012, a study reported that mifepristone decreased *HSP60* mRNA expression in HEp-2 cells infected with *Chlamydophila* and treated with IFNγ 4, suggesting that mifepristone might act as an inhibitor of HSP60.

Mifepristone is a steroid analog that acts as an antagonist to both the progesterone receptor (PR) and the glucocorticoid receptor (GR) 5. Currently, this compound is an approved for early pregnancy termination 6. However, several studies have indicated the potential of mifepristone to exert anti-tumor effects in specific types of cancers, including breast cancer 7, non-small cell lung cancer 8, prostate cancer 9, ovarian cancer 10, glioblastoma 11, and neuroblastoma 12. In a DMBA-induced mammary tumor model, mifepristone treatment resulted in a reduction in tumor incidence, multiplicity, and size 13, 14. Furthermore, in xenograft animal models, mifepristone inhibited the growth of ovarian cancer 10, prostate cancer 15, and glioblastoma xenograft 16. However, no studies have yet investigated the effects of mifepristone on HCC cells. Therefore, the present study aimed to investigate whether mifepristone could affect the viability of HCC cells by regulating the expression of HSP60.

In this study, we demonstrated the inhibitory effect of mifepristone on cell growth in HCC. Moreover, we have discovered a novel molecular mechanism involving HSP60, survivin, and GR, which contribute to the growth inhibitory effects in HCC cells. Additionally, the inhibitory effect of mifepristone on HCC and the proposed molecular effects have been validated using a xenograft model. These findings have significant implications and open up new treatment strategies for HCC.

## Materials and Methods

### Cell culture

The human HCC cell lines HepG2, Huh7 (authenticated by Genelabs Life Science Corp. using STR analysis in March 2017), as well as J7 17, were cultured in Dulbecco's modified Eagle's medium supplemented with 10% fetal bovine serum in a humidified incubator at 37°C with 5% CO_2_. Normal human hepatocytes (HH), purchased from ScienCell Research Laboratories (Carlsbad, CA), were cultured in Hepatocyte Medium following the manufacturer's instructions.

### MTT assay

Cells were plated in 48-well culture plates in the absence (0.05% DMSO only) or in the presence of mifepristone (10 μg/mL in 0.05% DMSO). The culture media, containing either 0.05% DMSO or mifepristone in 0.05% DMSO, were changed every four days. At different time points, cells were cultured in serum-free medium containing 0.5 mg/ml Thiazolyl Blue Tetrazolium Bromide (MTT, Sigma, St. Louis, MO). After 4 hours of incubation, solubilization solution (10% SDS in 0.01 M HCl) was added, and the reaction continued overnight. The cell proliferation index was measured by optical density at 570 nm with a reference wavelength at 650 nm using a plate reader.

### Immunoblot assay

Proteins were fractionated using SDS-PAGE and transferred to PVDF membranes (PerkinElmer, Boston, MA). Primary antibodies against Bcl-2 (Proteintech Group, Chicago, IL; Cat No.: 12789-1-AP), VDAC (Cell Signaling Technology, Beverly, MA; Cat No: 4661S), GR (Cell Signaling; Cat No: 47411S), cyclin E (Santa Cruz Biotechnology, Santa Cruz, CA; Cat No.: SC-481), CDK2 (Proteintech Group; Cat No.: 10122-1-AP), GAPDH (Millipore Corp.; Cat No.: MAB374), HSP60 (Proteintech Group; Cat No.: 66041-1-1g), PCNA (abcam; Cat No.: ab15497), and survivin (Proteintech Group; Cat No.: 10508-1-AP) were used for the immunoblot assay. Protein bands were quantified using Image Gauge software.

### RT-qPCR

The extracted RNA was used to perform cDNA synthesis using the MMLV Reverse Transcription kit (Life Technologies, Gaithersburg, MD). Real-time qPCR was performed using a 10 μL reaction mixture containing 1×SYBR Green reaction mixture, as well as forward and reverse primers. The ABI QuantStudio 3 Real-Time PCR system (Applied Biosystems, Foster City, CA) was employed to detect SYBR Green fluorescence. The primer sequence for HSP60 were as follows: forward, 5'-CACCGTAAGCCTTTGGTCATAAT-3', and reverse, 5'-CTTGACTGCCACAACCTGAAGA-3'. For survivin, the primer sequences were: forward, 5'-AGAGACCAGCAAGCCAAACTG-3', and reverse, 5'-GGCAATTGTGAGTTACTCTTTCCA-3'.

### Colony formation assay

Cells were cultured in 6-well plates with or without mifepristone (10 μg/mL in 0.05% DMSO) for a duration of 12 days. Subsequently, the cells were stained with crystal violet, and the resulting images were analyzed using Image J software.

### Flow cytometry

For cell cycle analysis, cells were cultured with or without mifepristone (10 μg/mL) for either 16 or 24 h. Subsequently, the cells were fixed in 70% ethanol at 4°C for 30 min. After removing the ethanol, the cells were suspended in a PBS solution containing 0.5% Triton X-100 and 25 μg RNase, and incubated at 37°C for 1 h. Propidium iodide (PI) was used to stain the cells, and flow cytometry was performed. For apoptosis analysis, the cells were treated with 5 μg/mL of mifepristone for 24 h because higher concentrations induced excessive cell death, leading to fewer discernible variations. After incubation, the cells were stained with fluorescein isothiocyanate (FITC)-conjugated annexin V and PI for 15 min at room temperature using the Annexin V:FITC Apoptosis Detection Kit (BD Pharmingen, San Diego, CA). Flow cytometry was then used to analyze the stained cells. For ΔΨm analysis, TMRE was used to label active mitochondria (TMRE Mitochondrial Membrane Potential Assay Kit; abcam, Cambridge, UK). Cells were cultured for 16 h with or without mifepristone (20 μg/mL), followed by incubation with TMRE (300 nM) for 25 min at 37°C. The cells were harvested with trypsin, and as a positive control, an inhibitor of mitochondrial oxidative phosphorylation called carbonyl cyanide 4-(trifluoromethoxy) phenylhydrazone (FCCP, 20 μM) was added to induce depolarization and eliminates ΔΨm. Signal detection and data analysis were performed using a FACSCalibur flow cytometer (Becton Dickinson, Franklin Lakes, NJ) and the Modfit LT program (Verity Software House, Brunswick, ME), respectively.

### Subcellular fractionation and immunoprecipitation assay

Cytosol and mitochondria were isolated from HCC cells or xenograft tumors using the Mitochondria Isolation Kit (Thermo Scientific) following the manufacturer's instructions. The isolated protein fractions were incubated overnight at 4°C with primary antibodies against survivin or GR. Prior to adding the immune complex, pre-reacted IgG antibodies (corresponding to the host species of the primary antibody) were incubated with Protein A/G-agarose beads (Santa Cruz Biotechnology) for 30 min at 4°C. Subsequently, the immunocomplexes were mixed with the beads and incubated for 1 h at 4°C. The proteins were eluted from beads using 2× SDS sample buffer and separated by SDS-PAGE for immunoblot analysis.

### MG132 treatment

After treating HCC cells with mifepristone (10 μg/mL) for 2 h (J7), 4 h (HepG2), and 18 h (Huh7), MG132 (10 μM) was added and incubated for 4 h (J7), 6 h (Huh7), and 19 h (HepG2).

### Plasmids and lentivirus infection

RNAi-coding plasmids targeting HSP60 (shHSP60) or GR (shGR), as well as an empty vector (pLKO.1), were obtained from Academia Sinica (National RNAi Core Facility Platform, Taiwan). Lentiviral particles carrying shHSP60, shGR, or a mock control were generated using the pPACKH1 Packaging Plasmid (SBI System Biosciences, Mountain View, CA), following the procedures described in our previous study 17.

### Subcutaneous xenograft models for mifepristone treatment

Male BALB/cAnN.Cg-*Foxnl^nu^*/CrlNarl mice, aged five weeks, were subcutaneously injected with Huh7 cells (2×10^6^/100 μL PBS). Once the xenograft tumors reached a size of 80~110 mm^3^, they were randomly divided into control and treatment groups. The control group received a vehicle (5% DMSO), while the treatment group received mifepristone (800 μg/each) nine times over a period of 12 days. Tumor volume (mm^3^) was calculated using the formula W×L×T (W: smallest diameter; L: longest diameter; T: thickness). All animal experiments were conducted in accordance with the approved standards of the Institutional Animal Care and Use Committee of Chang Gung University (CGU107-133), which holds a valid AAALAC certification.

### Immunohistochemistry

Paraffin-fixed sections were deparaffinized and subjected to staining for the detection of survivin, Ki-67, and active caspase-3. After blocking with 10% normal goat serum (Vector Labs, Burlingame, CA), primary antibodies against survivin, Ki-67 (Millipore Corp.; Cat No.: AB9260), and active caspase-3 (EnoGene Biotech, New York, NY; Cat No.: E11-0105L) were added and incubated overnight at 4°C. Subsequently, secondary antibodies (goat anti-rabbit, Vector Labs) were applied and incubated for 1 h at room temperature. The sections were then developed using diaminobenzidine solution for 5 min and counterstained with hematoxylin.

### TUNEL assay

Formalin-fixed xenograft tumors were embedded in paraffin, and DNA fragmentation in the paraffin-embedded tissue sections was assessed using the ApopTag^®^ Peroxidase* In situ* Apoptosis Detection Kit (Chemicon). Subsequently, the sections underwent protein digestion with proteinase K (20 μg/mL) and were treated with 3% hydrogen peroxide to block endogenous peroxidase activity. After incubation in equilibration buffer, the sections were exposed to the Working Strength TdT Enzyme in a humidified chamber at 37°C for 1 h. The reaction was halted using Stop/Wash buffer, and apoptotic nuclei were visualized by immunoperoxidase detection of digoxigenin-labeled DNA.

### Statistical analysis

Data are presented as the mean ± standard deviation (SD) of at least three independent experiments. Statistical significance between experimental and control groups was assessed using Student's t-test, and a p-value of < 0.05 was considered statistically significant.

## Results

### Mifepristone inhibits cell proliferation and promotes apoptosis in HCC Cells

**Figure 1:**
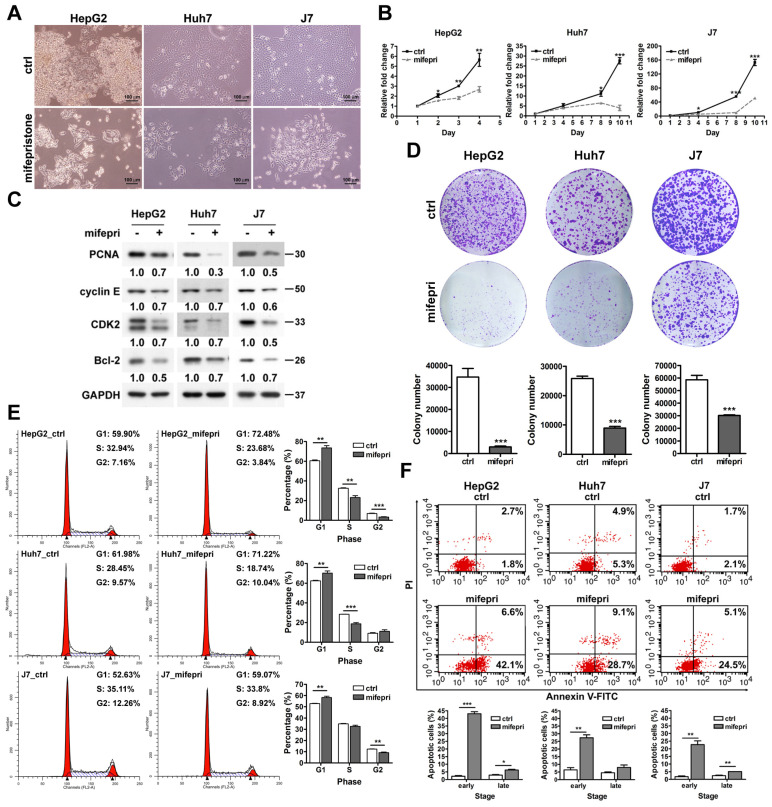
**Effect of mifepristone on the growth of HCC cells.** (A) Cell morphology of HCC cell lines (HepG2, Huh7, and J7) in the absence (ctrl) or presence of mifepristone (20 μg/mL) for 48 h. (B) MTT cell proliferation assay performed with or without mifepristone (mifepri, 10 μg/mL). (C) Immunoblot assay: HCC cells were treated with or without mifepristone (mifepri, 10 μg/mL) for 24 h. (D) Colony formation assay for HCC cells after mifepristone (mifepri) treatment. Top panel: representative image; Bottom panel: quantitative assessment of the number of colonies. (E-F) Cell cycle (E) and cell apoptosis (F) were analyzed using flow cytometry. The histogram displays the percentage of cells in each cell cycle phase (E) and the percentage of apoptotic cells in the early and late stages (F). Detailed conditions and procedures for mifepristone treatment are described in the Materials and Methods section. The data represent the mean ± SD from three independent experiments and were analyzed using Student's t-test. Statistical significance is indicated as *P < 0.05; **P < 0.01; ***P < 0.001 compared to the control (ctrl).

To evaluate the effect of mifepristone on HCC cells, we observed HepG2, Huh7, and J7 cell lines with and without mifepristone treatment using optical microscopy. The mifepristone-treated cells displayed reduced tight cell-to-cell connections and exhibited a more elongated morphology compared to the control group (Figure 1A). Additionally, mifepristone treatment not only inhibited HCC cell proliferation (MTT assay, Figure 1B) but also downregulated the expression of PCNA, a cell proliferation marker (Figure 1C). This effect was consistent with a noticeable decrease in the number of colonies formed by HCC cells upon mifepristone treatment (colony formation assay, Figure 1D). Furthermore, flow cytometry analysis revealed a significant increase in the proportion of cells in the G1 phase of the cell cycle in the mifepristone group compared to the control group across all three HCC cell lines (Figure 1E). Consistently, mifepristone treatment also reduced the expression of cyclin E/CDK2, a key regulator of G1/S phase transition (Figure 1C). Moreover, Annexin V-FITC/PI flow cytometry analysis showed that mifepristone induced both early and late apoptosis in HCC cells (Figure 1F). Notably, the percentage of cells in the early stage of apoptosis exhibited a significant increase upon mifepristone treatment (HepG2, from 2.1 ± 0.43% [control] to 43.0 ± 1.28% [mifepristone treatment]; Huh7, 6.4 ± 1.47% [control] to 27.4 ± 1.91% [mifepristone treatment]; J7, 1.8 ± 0.52% [control] to 22.7 ± 2.48% [mifepristone treatment]). Correspondingly, mifepristone treatment led to a reduction in the expression levels of the anti-apoptotic protein Bcl-2 in HCC cells (Figure 1C).

### Mifepristone promotes the release of HSP60 from mitochondria to the cytosol and reduces survivin expression in HCC cells

**Figure 2:**
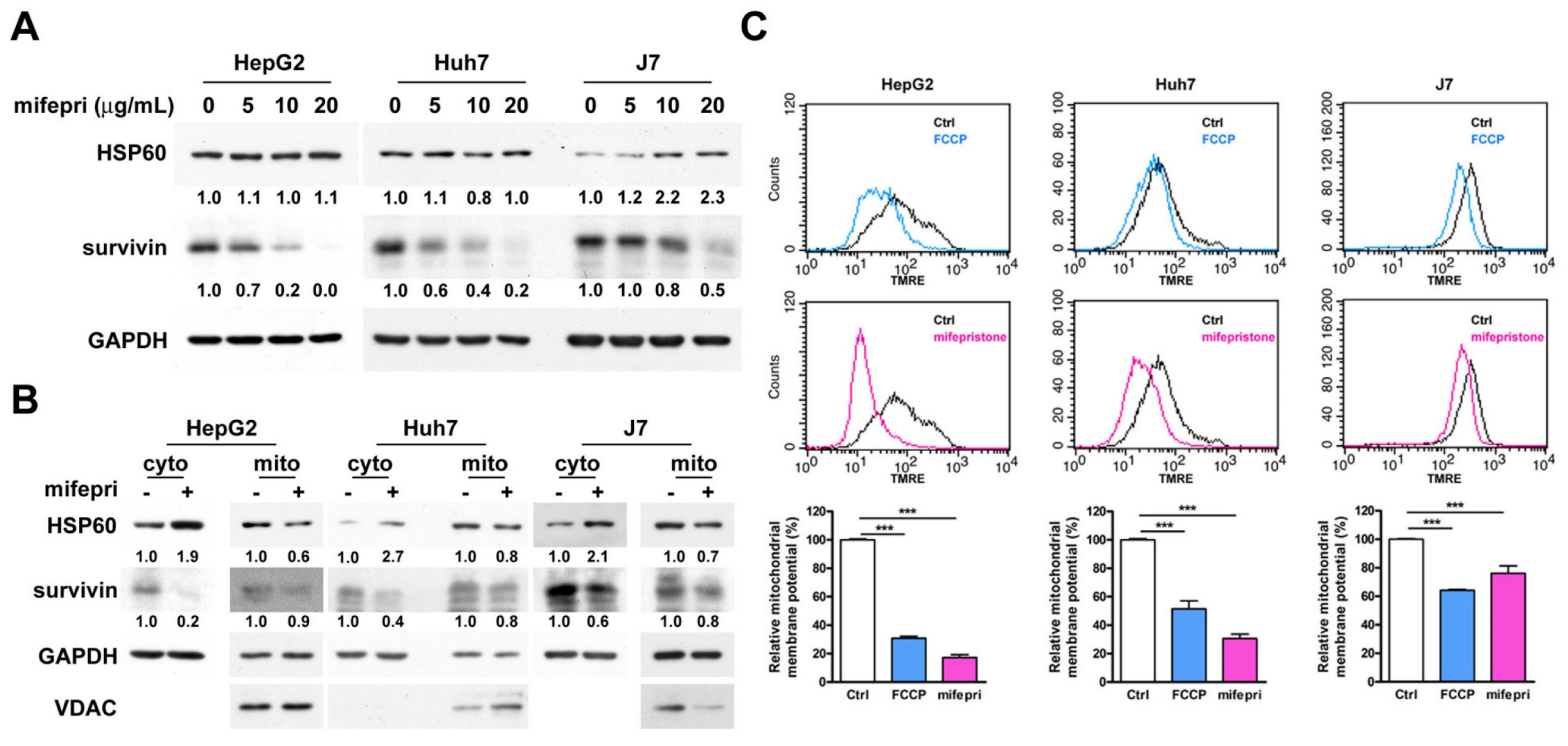
** Effect of mifepristone on HSP60 and survivin in HCC cells.** (A) Immunoblot assay was performed to detect the expression of HSP60 and survivin in HepG2, Huh7, and J7 cells treated with various concentrations of mifepristone (mifepri) for 72 h. GAPDH served as a loading control. The protein expression levels of genes in the group without mifepristone are used as references for calculating the fold differences among groups with different concentrations of mifepristone. (B) Cytosolic and mitochondrial fractions were extracted from cells with (+) or without (-) mifepristone (mifepri) treatment (10 μg/mL) for 48 h (J7) or 72 h (HepG2 and Huh7). Immunoblot assays were conducted to examine the levels of HSP60 and survivin. GAPDH served as a loading control, and Voltage-dependent anion channel (VDAC) was used as a mitochondrial marker. (C) Mitochondrial membrane potential was assessed through flow cytometry. HCC cells were stained with TMRE in the presence (red) or absence (black) of mifepristone (mifepri), while FCCP (blue) was used as a positive control. The top panel shows TMRE intensity distribution, and the bottom panel presents statistical results. The data present the mean ± SD from at least three independent experiments and were analyzed using Student's t-test. Statistical significance is indicated as ***P < 0.001 compared to the control (Ctrl).

Mifepristone inhibited the RNA expression level of HSP60 in HEp-2 cells 4. Additionally, HSP60 silencing decreased survivin expression in HCC cells 2. Therefore, we hypothesized that mifepristone could affect the expression of HSP60 and survivin in HCC cells. To test this hypothesis, total RNA and proteins were extracted from HCC cells treated with different concentrations (0, 5, 10, and 20 μg/mL) of mifepristone for RT-qPCR and immunoblot analysis. As shown in Figure S1, the RNA expression level of HSP60 slightly decreased in Huh7 and J7 cells after treatment with 20 μg/mL of mifepristone, while no significant difference was observed in HepG2 cells. Furthermore, the protein expression of HSP60 was only reduced in Huh7 cells when treated with 10 μg/mL of mifepristone. No decrease was observed in the other two HCC cells (Figure 2A). The RNA expression of survivin was significantly reduced in all three HCC cell lines treated with 20 μg/mL of mifepristone (Figure S1). After treatment with 10 μg/mL of mifepristone, the RNA expression of survivin decreased only in Huh7 cells (Figure S1). However, mifepristone decreased the protein expression of survivin in a dose-dependent manner in all HCC cell lines (Figure 2A), suggesting that mifepristone destabilizes the survivin protein. Considering that the HSP60-survivin interaction occurs in the cytosol of HCC cells but not in the mitochondria 2, the next step was to investigate whether the expression of HSP60 and survivin in the cytosol and(or) mitochondria could be affected by mifepristone. As shown in Figure 2B, treatment with mifepristone (10 μg/mL) reduced survivin levels in the cytosol and mitochondria, with a more pronounced decrease in the cytosol. Interestingly, HSP60 expression decreased in the mitochondria and increased in the cytosol in mifepristone-treated cells, implying that HSP60 was translocated from the mitochondria to the cytosol. The translocation of HSP60 from mitochondria to the cytosol could be attributed to the loss of mitochondrial membrane potential (ΔΨm), as a previous study reported a similar observation in lymphoma cells treated with mifepristone 18. To examine changes in ΔΨm, active mitochondria were labeled with tetramethylrhodamine, ethyl ester (TMRE), and flow cytometry analysis was performed. After exposure to mifepristone (20 μg/mL) for 16 h, ΔΨm was significantly reduced by 25.6% to 80.3% compared to control cells. As a positive control, FCCP-treated cells also showed a reduction in ΔΨm in HCC cells (Figure 2C). Additionally, 5-fluorouracil (5-FU), which decreased ΔΨm in HCC cells 19, also led to a reduction in mitochondrial HSP60 and an increase in cytosolic HSP60 in HCC cells (Figure S2). These findings suggest that mifepristone induces the release of HSP60 from mitochondria to the cytosol, likely due to the loss of ΔΨm.

### Effects of mifepristone on normal human hepatocytes

Mifepristone was tested using normal human hepatocytes (HH) to assess its potential effects. As shown in Figure S3A, the expression of HSP60 remained unaltered in HH cells when treated with concentrations of mifepristone ranging from 0 to 20 μg/mL. Moreover, while the expression of survivin showed slight changes (1.1 to 0.9-fold), these alterations did not achieve statistical significance. Additionally, no significant changes were observed in the cell cycle distribution (Figure S3B). Notably, mifepristone did induce early apoptosis in HH cells, resulting in a slight increase in the percentage (Figure S3C, from 4.5 ± 0.19% [control] to 10.7 ± 1.29% [mifepristone treatment]).

### HSP60 participates in mifepristone-induced survivin downregulation

**Figure 3:**
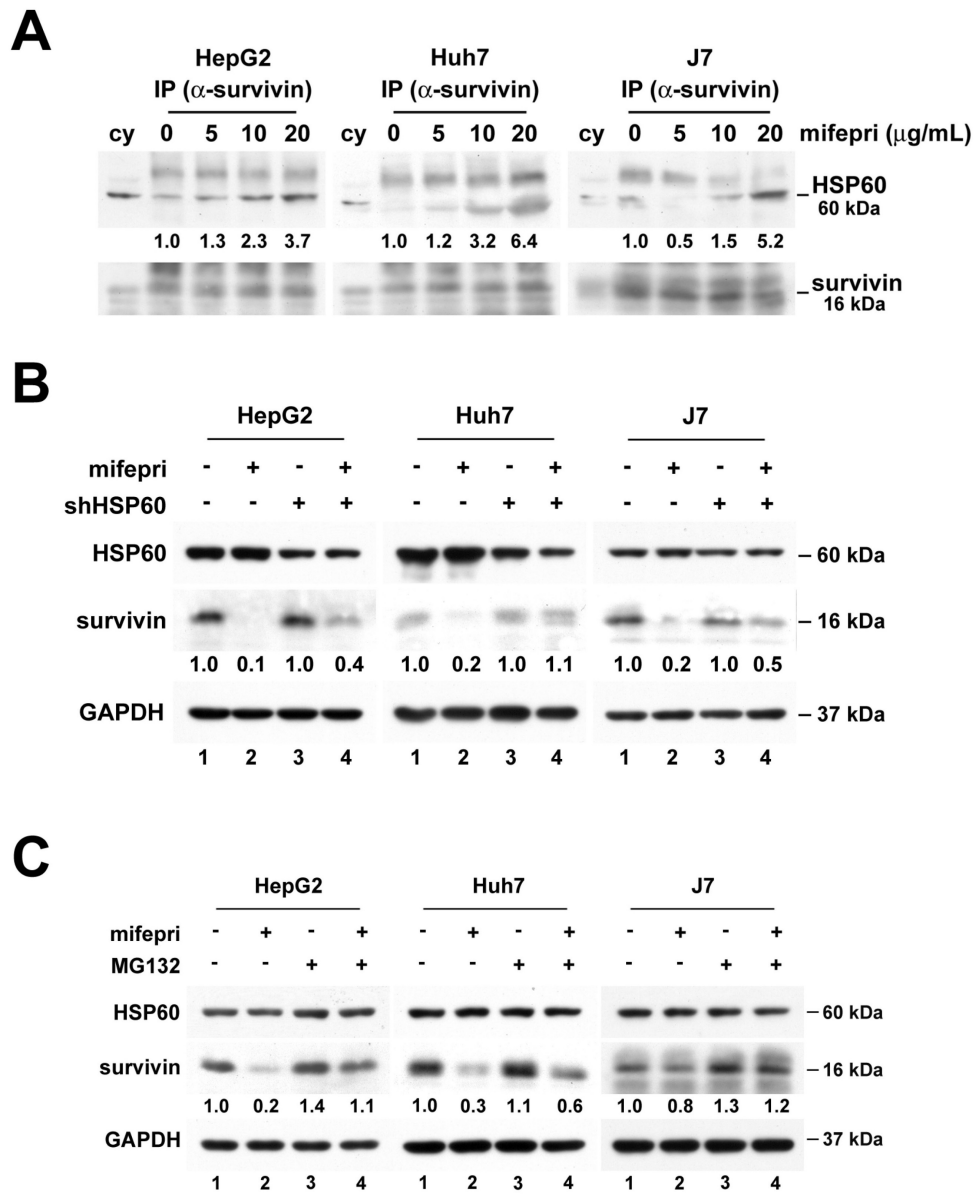
**Mifepristone-induced downregulation of survivin in HCC cells requires the involvement of HSP60.** (A) Cytosolic extracts from HepG2, Huh7, and J7 cells were incubated with increasing concentrations of mifepristone (mifepri; 5, 10, 20 μg/mL) for 4 h. The extracts were then subjected to immunoprecipitation using an anti-survivin antibody, followed by immunoblotting with an anti-HSP60 antibody. The protein expression levels of HSP60 in the group without mifepristone are used as references for calculating the fold differences among groups with different concentrations of mifepristone. (B) HepG2, Huh7, and J7 cells with (+) or without (-) HSP60 knockdown were treated with mifepristone (mifepri; 20 μg/mL) for 24 hours. The expression levels of HSP60, survivin, and GAPDH were detected using immunoblot assays. The survivin expression in lanes 1 and 3, without mifepristone treatment, serves as the reference for calculating the fold differences in the presence of mifepristone (lane 1 vs. lane 2 and lane 3 vs. lane 4). (C) Following treatment with (+) or without (-) mifepristone (mifepri), HepG2, Huh7, and J7 cells were treated with MG132 for 4-19 h to extract total protein. Immunoblot assays were performed to detect the expression of HSP60, survivin, and GAPDH. The survivin expression in lane 1 is used as the reference for calculating the fold differences among lanes 2, 3, and 4.

Since HSP60-survivin interactions occur in the cytosol of HCC cells 2, an immunoprecipitation assay was employed to investigate the effect of cytosolic HSP60-survivin interaction induced by mifepristone. The amount of cytosolic survivin in mifepristone-treated cells was lower than in untreated cells (Figure S4, lane 3 vs. lane 4), potentially affecting the detection results. Therefore, mifepristone (0, 5, 10, and 20 μg/mL) was directly incubated with cytosolic extracts for the immunoprecipitation assay. As shown in Figure 3A, mifepristone increased the interaction of cytosolic HSP60-survivin in a dose-dependent manner. Furthermore, to assess whether HSP60 plays a pivotal role in the mifepristone-induced downregulation of survivin expression, HSP60 was silenced by RNAi in HCC cells. As depicted in Figure 3B, mifepristone-induced downregulation of survivin expression was partially or completely restored in cells with HSP60 silencing (lane 2 vs. lane 4; 0.1 vs. 0.4 [HepG2], 0.2 vs. 1.1 [Huh7], 0.2 vs. 0.5 [J7]). Survivin expression levels are known to decrease rapidly during the G1 phase of the cell cycle through the ubiquitin-proteasome pathway 20. Given that mifepristone induces cell cycle arrest at the G1 phase accompanied by decreased levels of survivin (Figures 1E and 2A), we investigated whether mifepristone reduces survivin expression through proteasome-dependent degradation. After treating HCC cells with mifepristone for 2-18 h, a proteasome inhibitor, MG132, was employed. As demonstrated in Figure 3C, the downregulation of survivin expression by mifepristone in HCC cells was partially or entirely reversed after MG132 treatment (lane 2 vs. lane 4; 0.2 vs. 1.1 [HepG2], 0.3 vs. 0.6 [Huh7]; 0.8 vs. 1.2 [J7]). These results indicate that mifepristone negatively regulates survivin expression through proteasome-mediated degradation.

### GR participates in mifepristone-mediated survivin degradation

**Figure 4:**
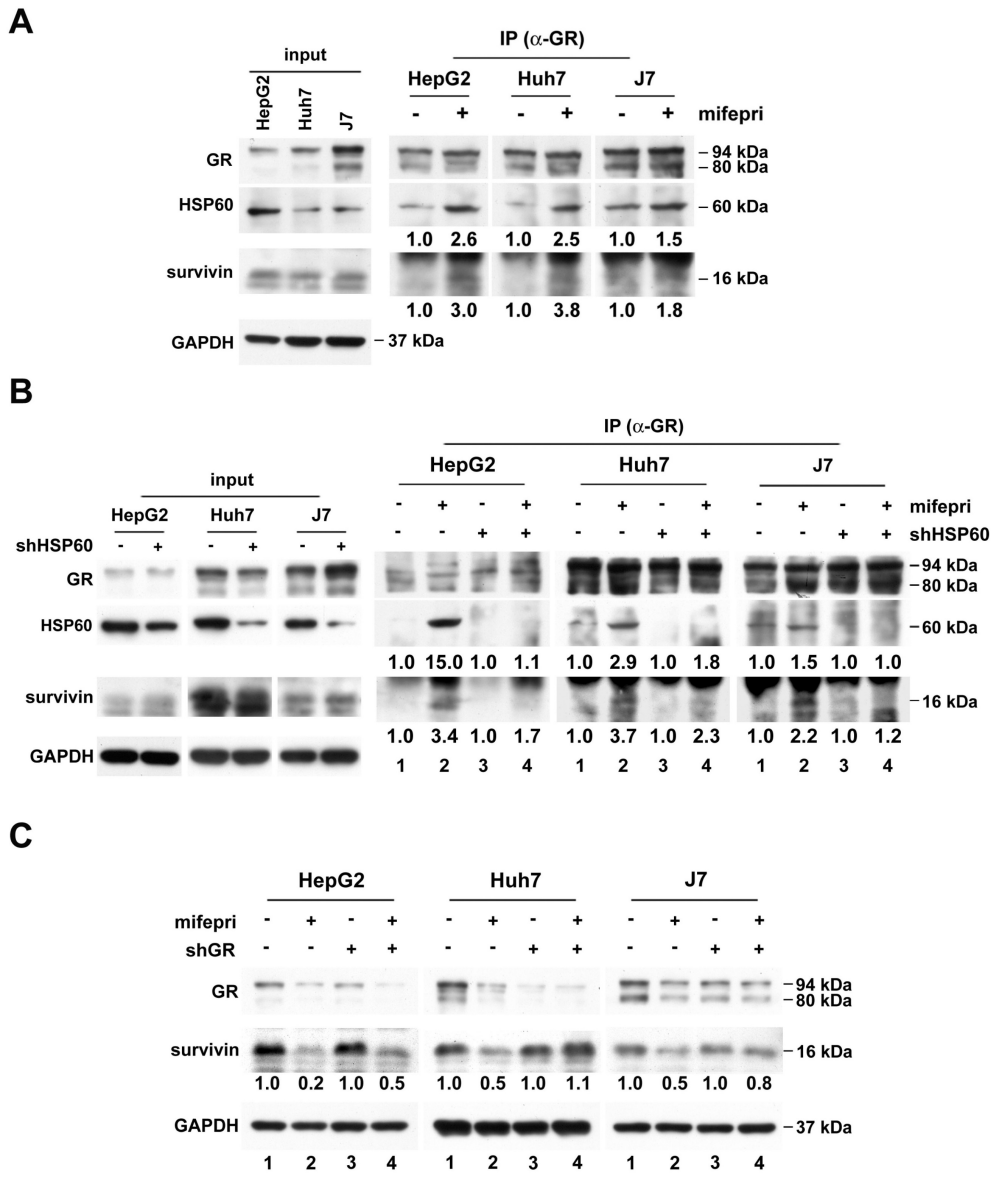
**GR participates in mifepristone-trigged survivin degradation in HCC cells.** (A-B) Immunoprecipitation was performed using cytosolic extracts from untreated HCC cells (A) and cells with (+) or without (-) shHSP60 expression (B). After incubation of cytosolic extracts with (+) or without (-) mifepristone (mifepri) for 4 h, they were immunoprecipitated with anti-GR antibodies to examine the amounts of GR, HSP60, and survivin by immunoblot. The left panels (A and B) are represent input controls for cytosolic extracts. (C) Total protein was extracted from GR silencing (shGR) HCC cells with or without mifepristone (mifepri) treatment for the detection of GR and survivin by immunoblot. GAPDH served as a loading control. The lane in the group without mifepristone treatment serves as the reference for calculating the fold differences in the presence of mifepristone.

Mifepristone, an antagonist of both PR and GR, suggests the possible involvement of both steroid receptors in mifepristone-induced survivin degradation. However, we excluded PR due to its extremely low expression in both tumor and non-tumor tissues of HCC patients 21. Instead, we focused on GR, which is overexpressed in HCC tissues 22. To explore whether GR also participates in the mifepristone-mediated enhancement of the HSP60-survivin interaction, we performed immunoprecipitation of cytosolic proteins with an anti-GR antibody in the presence or absence of mifepristone. As shown in Figure 4A (right panel; middle and bottom), in the absence of mifepristone (-), only HSP60, and not survivin, was detected in GR immunoprecipitates. However, in the presence of mifepristone (+), increased signals of both HSP60 and survivin were detected.

Next, we investigated whether GR binds to survivin through HSP60. Cytosolic proteins extracted from HCC cells with or without HSP60 silencing were used for immunoprecipitation using an anti-GR antibody, followed by survivin detection. Figure 4B demonstrates that the mifepristone-enhanced GR-survivin interaction was abolished in HSP60-silenced cells (right panel; bottom, lane 2 vs. lane 4 for each cell line). Furthermore, to assess the role of GR in mifepristone-mediated survivin degradation, we knocked down GR in HCC cells. As shown in Figure 4C, the reduced expression of survivin induced by mifepristone was mildly or entirely restored in cells with GR silencing (Figure 4C, middle, lane 2 vs. lane 4; 0.2 vs. 0.5 [HepG2], 0.5 vs. 1.1 [Huh7]; 0.5 vs. 0.8 [J7]). Overall, the results indicate that GR is involved in mifepristone-mediated survivin degradation, possibly due to its enhanced interactions with HSP60 and survivin.

### Inhibition of tumor growth by mifepristone in a mouse xenograft model

**Figure 5:**
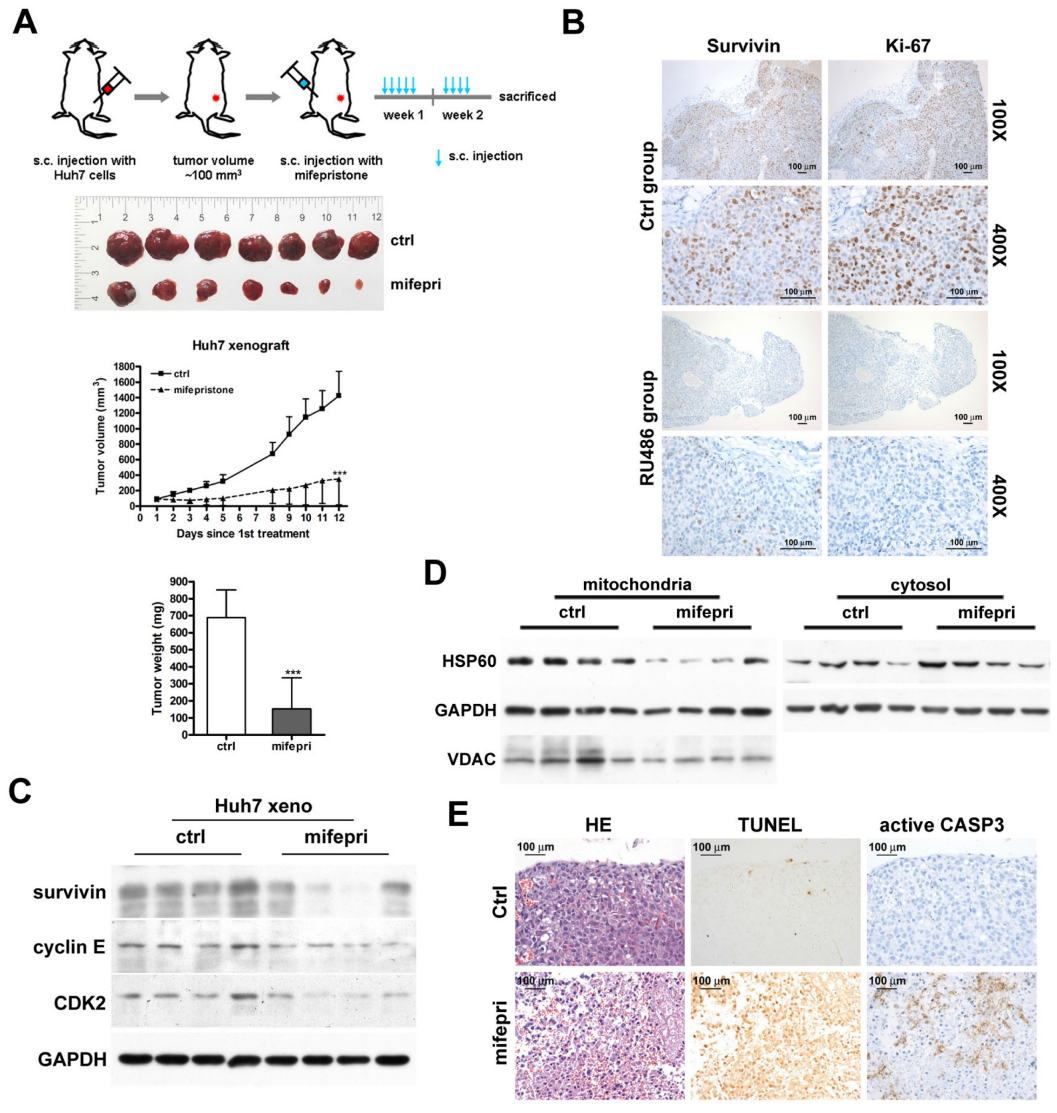
**Suppression of tumor growth by administration of mifepristone in a Huh7 xenograft mouse model.** (A) Top: Schematic representation of the establishment of Huh7 xenograft mouse models and treatment with mifepristone. Middle: Final xenograft tumors and tumor volume for the control (ctrl) and mifepristone (mifepri) groups. Bottom: Tumor weight for the control (ctrl) and mifepristone (mifepri) groups. “***”, *P* < 0.001. (B) Immunohistochemical images showing survivin and Ki-67 (a marker for cell proliferation) staining at magnifications of 100× and 400×. (C) Immunoblot assay. Protein expression levels of survivin, cyclin E, and CDK2 in the control (ctrl) and mifepristone (mifepri) groups. (D) Changes in HSP60 expression in mitochondria and cytosol of xenograft tumors from the control (ctrl) and mifepristone (mifepri) groups. GAPDH is used as a loading control, and VDAC is a mitochondrial marker. (E) H&E staining (HE), TUNEL assay, and immunohistochemical staining of active caspase-3 (CASP3) for xenograft tumors.

To confirm the inhibitory effect of mifepristone on HCC growth *in vivo*, we employed the Huh7 xenograft model. Figure 5A (top) provides an overview of the experimental procedure. After administering mifepristone to the mice nine times over 12 days, the tumor volume in the mifepristone group was significantly smaller compared to the control group (Figure 5A). Moreover, the mifepristone group exhibited a marked reduction in tumor weight (152.9 ± 182.37 mg) compared to the control group (688.8 ± 162.98 mg) (Figure 5A, bottom). Immunohistochemistry analysis revealed a decrease in the number of survivin-positive cells in mifepristone-treated xenograft tumors compared to the control group (Figure 5B). The mifepristone group exhibited a lower expression level of survivin, as determined by immunoblot assay, compared to the control group (Figure 5C). Furthermore, the expression level of mitochondrial HSP60 was lower in the mifepristone group compared to the control group (Figure 5D, left), while the expression level of cytosolic HSP60 was higher (Figure 5D, right). The density and number of Ki-67-positive cells were lower in the mifepristone group compared to the control group (Figure 5B, right). Additionally, the mifepristone group demonstrated lower expression levels of cyclin E and CDK2 compared to the control group (Figure 5C). The TUNEL assay revealed a higher number of apoptotic cells in the mifepristone group compared to the control group (Figure 5E, middle). Moreover, within the apoptotic region of the mifepristone group, the number of active caspase-3 (an apoptosis indicator)-positive cells was also higher (Figure 5E, right).

## Discussion

Clinical trials have indicated the potential efficacy of mifepristone against certain types of advanced cancer 23, 24; however, its impact on HCC has not been previously investigated. In this study, we discovered that mifepristone inhibits HCC cell growth, potentially through increased formation of the GR-HSP60-survivin complex in the cytosol, leading to survivin degradation. HSP60 and survivin in the cytosol are involved in mifepristone-induced apoptosis. However, the functions of cytosolic HSP60 can vary, exhibiting either pro-survival or pro-death properties 25, 26. For instance, in HeLa cells, cytosolic HSP60 has been shown to enhance the TNF-α-mediated activation of the IKK/NF-κB survival pathway, indicating a pro-survival role 25. Conversely, during staurosporine-induced apoptosis in Jurkat cells, increased expression of cytosolic HSP60 (resulting from its release from mitochondria) was observed, suggesting a pro-apoptotic function 27. Similarly, in the context of mifepristone-induced apoptosis in HCC cells, we observed a decrease in mitochondrial HSP60 expression, an increase in cytosolic HSP60 expression, and a reduction in ΔΨm. In 2007, Chandra et al. proposed that cytosolic HSP60 serves as a pro-apoptotic factor, requiring its release from mitochondria 28. Thus, our findings on mifepristone-induced apoptotic HCC cells further support the theory of cytosolic HSP60 (released from mitochondria) functioning as a pro-apoptotic factor.

Survivin, similar to HSP60, has been observed to translocate from the mitochondria to the cytosol in response to external stimuli, as demonstrated in previous research using INS-1 cells expressing mitochondrial survivin 29. However, in our study, we did not observe this phenomenon in HCC cells treated with mifepristone (Figure 2B). Additionally, previous research has shown that survivin expression increases during the G2/M phase of the cell cycle and undergoes rapid degradation during the G1 phase 20. Consistently, mifepristone treatment induced cell cycle arrest at the G1 phase, accompanied by a significant decrease in survivin levels in HCC cells. There is a growing body of evidence supporting the inhibition of tumor cell growth through the silencing of survivin in various types of cancers 30-33. Therefore, the reduction of survivin levels by mifepristone may contribute to the inhibition of growth in HCC cells.

The loss of ΔΨm has been widely recognized as a key event in apoptosis, triggering the release of cytochrome *c* and activation of the caspase cascade 34. In 1999, Samali et al. demonstrated that staurosporine-induced apoptosis resulted in both ΔΨm loss and the release of HSP60 from the mitochondrial membrane space into the cytoplasm 27. Similarly, we observed a similar phenomenon in mifepristone-induced apoptotic HCC cells, indicating a dual effect of mifepristone: ΔΨm loss leading to the leakage of HSP60 into the cytosol and an increase in the formation of the GR-HSP60-survivin complex, ultimately leading to survivin degradation.

**Figure 6:**
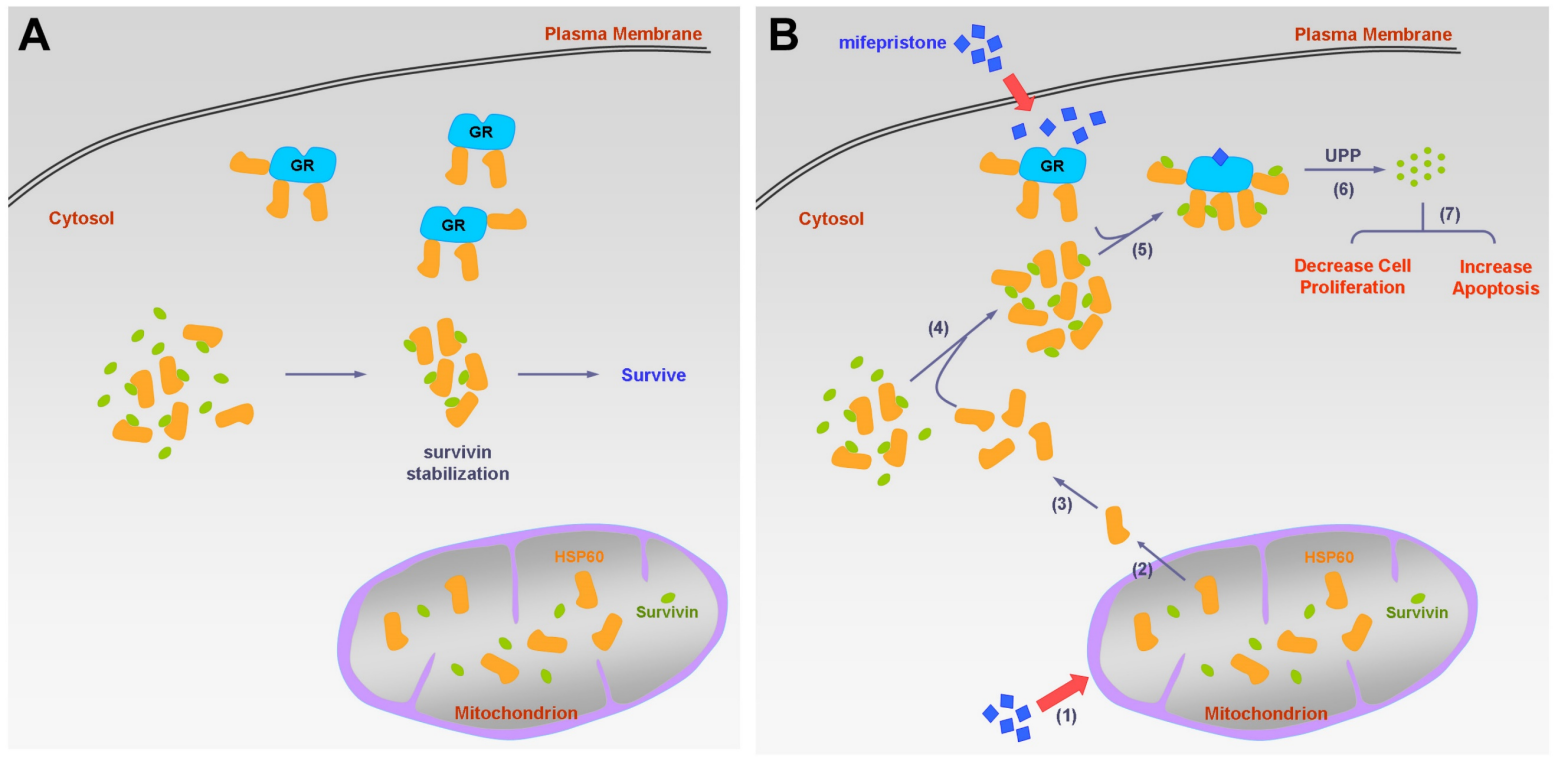
** Schematic diagram of the molecular mechanism on mifepristone in inhibiting HCC cell growth.** This figure illustrates the roles of GR, HSP60, and survivin in HCC cells in the presence and absence of mifepristone, showcasing the molecular mechanism behind its inhibitory effects. (A) Under normal physiological conditions, HSP60-GR and HSP60-survivin complexes are localized in the cytosol. HSP60 binds to survivin, thereby stabilizing survivin for cell survival. (B) In the presence of mifepristone, it not only binds to GR but also reduces the activity of ΔΨm, leading to the release of HSP60 from mitochondria to the cytosol (steps 1 to 2). The accumulation of cytosolic HSP60 enhances the formation of HSP60-survivin complexes, facilitating interaction with GR (steps 3 to 5). Subsequently, survivin is degraded through the ubiquitin-proteasome pathway (UPP) (step 6). Ultimately, this process results in decreased cell proliferation and increased apoptosis (step 7).

In this study, we made several important findings regarding the molecular mechanisms of mifepristone in inhibiting HCC growth. Firstly, we demonstrated that under normal physiological conditions (without mifepristone treatment), cytosolic survivin binds to HSP60 but not to GR. However, in the presence of mifepristone, GR-survivin binding occurs, and its interaction requires the involvement of HSP60. Additionally, we observed that lower concentrations of mifepristone (10 μg/mL) led to a decrease in survivin protein levels without affecting survivin RNA levels, indicating post-translational degradation of survivin. Furthermore, mifepristone treatment resulted in a reduction in both survivin and GR expression levels (Figure 4C, lane 1 vs. lane 2 for each cell line). However, when GR was silenced in HCC cells, the decreased expression of survivin induced by mifepristone was partially or completely restored, suggesting that the presence of GR is necessary for mifepristone-mediated reduction of survivin. Interestingly, we also observed that HCC cells with higher endogenous GR expression (J7 cells) were more sensitive to the growth inhibitory effects of mifepristone compared to other HCC cells (Figure S5). This suggests that both the involvement and abundance of GR play a role in mifepristone-mediated growth inhibition. It is worth noting that the reduction in GR expression induced by mifepristone is expected to occur later than the reduction of survivin. Overall, Figure 6 provides a summary of the molecular mechanisms by which mifepristone inhibits HCC growth, highlighting the involvement of GR, HSP60, and survivin in this process.

Finally, the HCC xenograft animal model confirmed the growth inhibitory effects of mifepristone and the altered abundance of HSP60 and survivin. An increase in apoptosis was detected in the tumors of mice receiving mifepristone, accompanied by an increase in active caspase-3 and a decrease in Ki-67 expression. This preclinical study, demonstrating the therapeutic potential of mifepristone against HCC, will be valuable for future clinical trials.

## Supplementary Material

Supplementary figures and tables.
