# Supplementary material for: Mifepristone inhibits hepatoma growth by enhancing the GR-HSP60-survivin interaction to facilitate survivin degradation
Source: J Cancer. 2023 Sep 18;14(16):3066–77. doi: 10.7150/jca.86611 (PMC10583585; doi:10.7150/jca.86611)
Supplement: Supplementary file 1 — Supplementary figures and tables. [file jcav14p3066s1.pdf]

Supplementary Figures and Figure legends

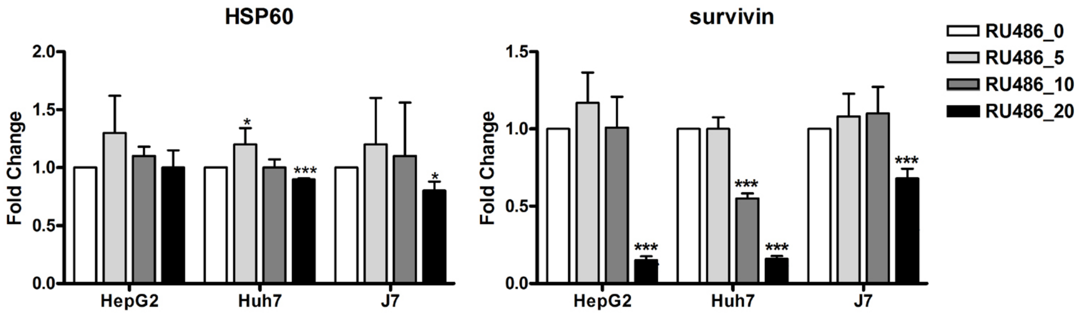

Figure S1. RNA expression of HSP60 and survivin in HCC cells upon treatment with RU486. HepG2, Huh7 and J7 were treated with different concentrations of RU486 (0, 5, 10, and 20 μg/mL). Detection of HSP60 and survivin by RT-qPCR. Data are the mean ± SD of at least three independent experiments. "*", $P < 0.05$; "**", $P < 0.01$; "***", $P < 0.001$.

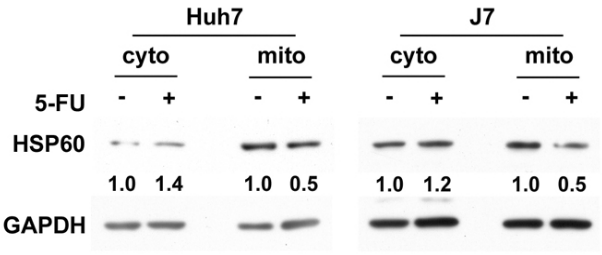

Figure S2. HSP60 expression levels in cytosol and mitochondria in HCC cells upon 5-FU treatment. The cytosolic and mitochondrial fractions extracted from 5-FU-treated Huh7 and J7 cells were used to detect HSP60 by immunoblot.

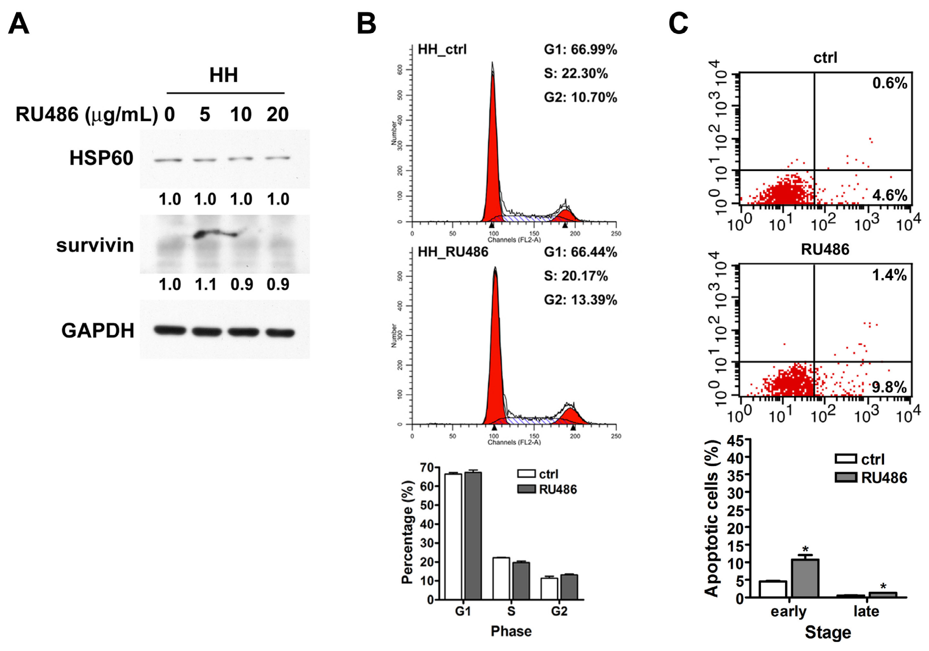

Figure S3. Effects of RU486 in normal human hepatocytes. (A) Normal human hepatocytes (HH) were treated with different concentrations of RU486. Detection of HSP60 and survivin by immunoblot assay. GAPDH is a loading control. (B-C) Cell cycle (B) and cell apoptosis (C) were determined by flow cytometry analysis. The histogram shows the percentage of cells in each phase of the cell cycle (B) and the percentage of apoptotic cells in the early and late stages (C). "*", $P < 0.05$.

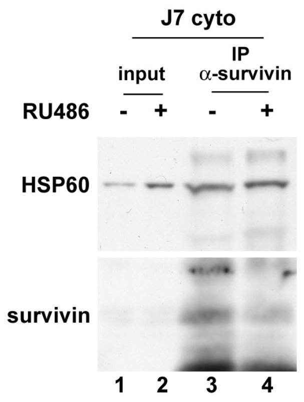

Figure S4. The amount of cytosolic survivin in HCC cells treated with RU486 was reduced after immunoprecipitation using an anti-survivin antibody. Cytosol proteins were extracted from J7 cells treated with (+) or without (-) RU486 for 24 h. Immunoprecipitation using anti-survivin antibody for the detection of HSP60 and survivin by immunoblot.

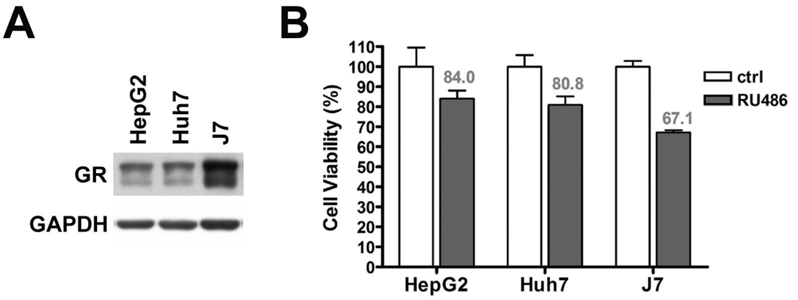

Figure S5. High endogenous GR expression in HCC cells is sensitive to the effects of RU486. (A) Expression levels of endogenous GR in HepG2, Huh7 and J7 cells were detected by immunoblot. (B) Cell viability in HCC cells with (RU486) or without (ctrl) RU486 treatment was examined by MTT assay.
